# Comparison of Bacterial DNA Profiles in Mid-Trimester Amniotic Fluid Samples From Preterm and Term Deliveries

**DOI:** 10.3389/fmicb.2020.00415

**Published:** 2020-03-24

**Authors:** Lisa Stinson, Maria Hallingström, Malin Barman, Felicia Viklund, Jeffrey Keelan, Marian Kacerovsky, Matthew Payne, Bo Jacobsson

**Affiliations:** ^1^Division of Obstetrics and Gynaecology, Faculty of Health and Medical Sciences, The University of Western Australia, Crawley, WA, Australia; ^2^Women and Infants Research Foundation, Subiaco, WA, Australia; ^3^Department of Obstetrics and Gynecology, Sahlgrenska University Hospital, Gothenburg, Sweden; ^4^Department of Obstetrics and Gynecology, Institute of Clinical Sciences, Sahlgrenska Academy, University of Gothenburg, Gothenburg, Sweden; ^5^Food and Nutrition Science, Department of Biology and Biological Engineering, Chalmers University of Technology, Gothenburg, Sweden; ^6^Stockholm South General Hospital, Stockholm, Sweden; ^7^Department of Obstetrics and Gynecology, Faculty of Medicine in Hradec Kralove, University Hospital Hradec Kralove, Charles University, Hradec Kralove, Czechia; ^8^Biomedical Research Centre, University Hospital Hradec Kralove, Hradec Kralove, Czechia; ^9^Department of Genetics and Bioinformatics, Area of Health Data and Digitalization, Institute of Public Health, Oslo, Norway

**Keywords:** amniotic fluid, bacteria, 16S rRNA, preterm birth, microbial invasion of amniotic cavity

## Abstract

Infection and inflammation are well recognized causes of spontaneous preterm delivery (PTD) (<37 gestational weeks) and adverse infant outcomes. To date, there has been very little investigation into bacterial communities in amniotic fluid using next generation sequencing technology. In particular, it is important to characterize amniotic fluid bacterial profiles in complicated pregnancies as well as in asymptomatic women to identify predictive bacterial DNA signatures. Here, 1198 mid-trimester amniotic fluid samples from a cohort of Swedish women undergoing mid-trimester genetic amniocentesis were screened for bacterial DNA using qPCR protocols specifically designed to reduce the impacts of reagent contamination and human DNA mispriming. The majority of samples were devoid of detectable bacterial DNA; however, approximately a fifth of the cohort (19.9%) were 16S rRNA gene positive in duplicate screening. Among these, nine women had a spontaneous PTD. These nine women were matched with 18 healthy women with a delivery at term. We used PacBio SMRT technology, coupled with appropriate negative extraction and PCR controls, to sequence the full-length 16S rRNA gene in this subset of 27 women. The amniotic fluid samples contained low-abundance and low-diversity bacterial DNA profiles. Species typically associated with spontaneous PTD were absent. We were not able to identify any differences in the amniotic fluid bacterial DNA profiles of women with a subsequent spontaneous PTD compared to women who delivered at term. The findings suggest that, in a minor proportion of pregnancies, DNA from non-pathogenic bacteria may be present in the amniotic fluid far earlier than previously reported. Early detection of bacterial DNA in the amniotic fluid was, in this study, not associated with spontaneous PTD.

## Background

The existence of a fetal or *in utero* microbiome (an ecosystem of interacting microorganisms) in normal pregnancy remains controversial (Collado et al., 2016; Stinson et al., 2016; de Goffau et al., 2019). Numerous studies have investigated bacterial colonization of the amniotic fluid in both complicated and healthy pregnancies using culture-dependant and culture-independent techniques. These studies have reported a diverse range of results, with some finding that amniotic fluid was devoid of bacteria (Rowlands et al., 2017), some finding that all amniotic fluid samples contain bacteria (Collado et al., 2016; Urushiyama et al., 2017; Wang et al., 2018; Zhu et al., 2018), and yet others reporting a combination of positive and negative samples (Watts et al., 1992; Mandar et al., 2001; Bearfield et al., 2002; Mendz et al., 2013; Cobo et al., 2017; Kayem et al., 2018; Lim et al., 2018; Morimoto et al., 2018; Stinson et al., 2019a). While pathogenic colonization of the amniotic fluid has been well-documented and thoroughly studied in the context of spontaneous preterm delivery (PTD; <37 gestational weeks) and fetal infection (DiGiulio, 2012; Mendz et al., 2013; Stinson and Payne, 2019; Thorell et al., 2019), little is known about the nature, origins, and significance of any endemic amniotic fluid bacteria in normal pregnancies. In particular, the timing and stability of such colonization remains largely unexplored. Such information is important, as bacterial populations in the amniotic fluid are likely to influence fetal skin and gut colonization (via swallowing of the amniotic fluid which begins at the end of the first trimester) which could have important implications for the development and maturation of the fetal immune system.

To date, there has been limited characterization of bacterial populations in amniotic fluid from uncomplicated pregnancies using next generation sequencing (Collado et al., 2016; Urushiyama et al., 2017; Kayem et al., 2018; Lim et al., 2018; Wang et al., 2018; Zhu et al., 2018; Stinson et al., 2019a). Unfortunately, most previous studies are complicated by the fact that appropriate and stringent extraction and PCR controls were not used. None of these studies attempted to eliminate reagent-derived contamination using laboratory-based techniques, such as those previously described by our group (Stinson et al., 2018). This makes it difficult to make conclusions on the true level of bacterial DNA in their samples. Apart from being inadequately controlled, these studies have all relied on short 16S rRNA gene amplicons with different variable regions for their sequencing. While such an approach is somewhat useful for broad community profiling without detailed taxonomic information, it is not able to give accurate species level assignment (Martinez-Porchas et al., 2016). Additionally, the selection of the region of the 16S rRNA gene to be sequenced can introduce bias into the results (Cruaud et al., 2014; Tremblay et al., 2015). Furthermore, some of these studies used QIIME v1 for operational taxonomic unit (OTU) clustering and taxonomic assignment, a platform which has been shown to give inaccurate taxonomic assignments and to inflate the number of OTUs in a sample when the default settings are used (Edgar, 2017).

The existence of an amniotic fluid microbiome is contentious; however, there is some evidence to suggest that bacterial DNA is commonly found in amniotic fluid at delivery (Collado et al., 2016; Urushiyama et al., 2017; Wang et al., 2018; Stinson et al., 2019a). It is not known when in gestation bacteria may first be able to access the amniotic space. Apart from three 16S rRNA gene sequencing studies (Urushiyama et al., 2017; Kayem et al., 2018; Zhu et al., 2018), studies of microbial profiles in mid-trimester amniotic fluid to date have largely relied on bacterial culture and targeted real-time PCR, and have primarily focused on identifying pathogenic bacteria involved in the etiology of spontaneous PTD (Watts et al., 1992; Bearfield et al., 2002; Payne et al., 2014; Rowlands et al., 2017; Morimoto et al., 2018). High quality data on the full microbial ecology of mid-trimester amniotic fluid is lacking, making it difficult to speculate on the extent to which bacterial DNA is found in amniotic fluid at this time point. Importantly, by comparing bacterial profiles in amniotic fluid samples from women with a spontaneous PTD and those with a term delivery, we may be able to identify a predictive microbiome signature of high-risk pregnancies.

There is currently a need for robust, well-controlled studies of the bacterial content of amniotic fluid in normal and high-risk pregnancies, particularly one which employs full-length 16S rRNA gene sequencing and appropriate measures to reduce external contamination so that low biomass microbiota can be described with confidence. In the present study, we characterized the bacterial DNA profiles of amniotic fluid collected during mid-trimester genetic amniocentesis from women with no clinical evidence of infection that went on to deliver at term or by spontaneous PTD.

## Materials and Methods

### Cohort Selection

**FIGURE 1:**
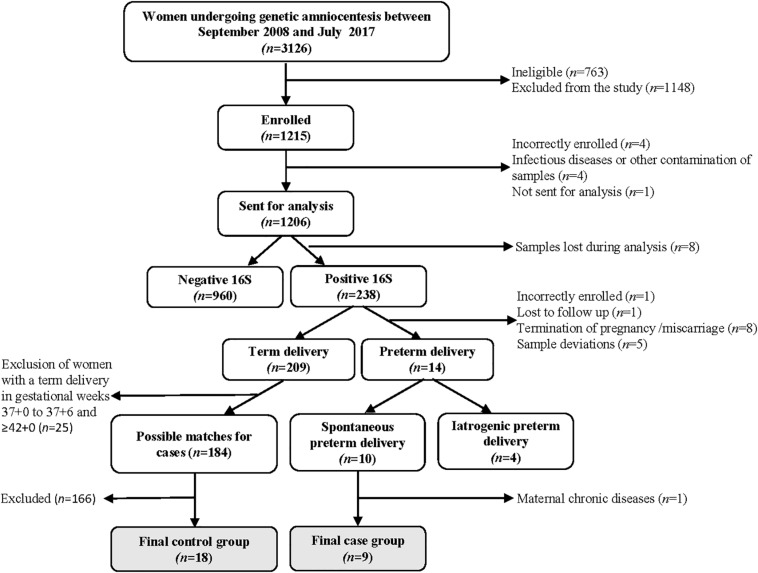
Flow chart of the cohort and selection of cases and controls.

Between September 2008 and July 2017, 3126 women underwent a mid-trimester genetic amniocentesis at Sahlgrenska University Hospital/Östra, Gothenburg, Sweden (Figure 1). Amniocentesis was performed per clinical indications such as advanced maternal age, anxiety, abnormal first-trimester combined screening or family history of chromosomal abnormalities or genetic diseases. Women ≥18 years of age with a viable singleton pregnancy were eligible. Women with multiple pregnancy, infectious diseases (such as HIV or hepatitis B), who had known or suspected fetal malformations as well as women undergoing amniocentesis during times when study samples could not be collected were considered ineligible (*n* = 763). Women who declined participation, who had language difficulties and therefore could not provide informed consent in Swedish, or if an insufficient amount of fluid was retrieved during amniocentesis, were considered excluded (*n* = 1148). The remaining 1215 women were enrolled into the study. 16S rRNA gene data was retrieved from 1198 women, of which 238 women tested positive in the qPCR screen. Among these, nine healthy women had a spontaneous PTD and they were matched with women who delivered in gestational weeks 38^+0^ to 41^+6^ to a 1:2 ratio using the immediately preceding and following women in the cohort (Figure 1), according to the following criteria:

1. Parity (primiparous or multiparous),
2. BMI (matched according to WHO definitions of BMI categories: underweight (<18.5); normal weight (18.5–24.9); pre-obesity (25.0–29.9); and obesity class I–III (≥30.0),
3. Smoking (yes/no),
4. IVF (yes/no),
5. Ethnicity [categorized according to Burchard et al. (2003): Black or African American (Africans); White (Caucasians); and Asian].

### Sample Collection

Three milliliter of amniotic fluid was collected for research purposes (in addition to that collected for diagnostic use) during mid-trimester genetic amniocentesis performed at 14–20 gestational weeks at Sahlgrenska University Hospital/Östra, Gothenburg, Sweden. Amniotic fluid samples were immediately stored at 4–8°C. The samples were centrifuged at 12,000 × *g* for 20 min at 4°C to pellet the cells. Pellets were resuspended in 1 mL sterile 1× PBS and frozen at −80°C until processing. Samples were initially shipped on dry ice to the Institute of Molecular Biomedicine, Comenius University, Bratislava, Slovakia, where 200 μL of cell pellet resuspension from each sample was aspirated under aseptic conditions for downstream analyses unrelated to the current study. These samples were again frozen at −80°C and shipped on dry ice to the Division of Obstetrics and Gynaecology, The University of Western Australia, Crawley, WA, Australia, for microbial profiling analyses used in the current study. Other samples were sent directly (on dry ice) to the Division of Obstetrics and Gynaecology, The University of Western Australia, Crawley, WA, Australia from Gothenburg, Sweden. These had thus not been thawed. There was no difference in the downstream 16S rRNA gene qPCR results between the samples that were shipped initially to Slovakia and those that were shipped directly to Australia.

### DNA Extraction

Amniotic fluid suspensions were centrifuged at 40,000 × *g* for 5 min at 4°C to pellet cells. Cell pellets were resuspended in 353 μL Buffer MBL/RNase A solution (QIAGEN) and DNA was extracted using a QIAGEN MagAttract Microbial DNA Kit on the KingFisher Flex platform as per manufacturer’s instructions. One blank extraction control (EC) was included in the center of each 96 deep-well plate.

### qPCR Screening

The 1198 samples were screened for bacterial DNA using a custom Taqman qPCR protocol, adapted from Yang et al. (2002) with a new forward primer to improve coverage of *Ureaplasma* spp. and *Mycoplasma* spp. The V6 region of the 16S rRNA gene was amplified in 20 μL reactions containing 6 μL of template or water (negative template control), 1× TaqMan Fast Advanced Master Mix (Applied Biosystems), 0.9 μM each of the forward (917F 5′-GAATTGACGGGGRCCCGC-3′) and reverse (1033R 5′-TGCGGGACTTAACCCAACA-3′) primers, 0.25 μM of probe (5′-FAM-CACGAGCTGACGACARCCATGCA-TAMRA-3′), and 0.95 μL of water. Master mix solutions were treated with a PCR Decontamination Kit (ArcticZymes^®^), which consisted of a double-stranded DNase (dsDNase) and DTT (which helps to inactivate the dsDNase), as previously described (Stinson et al., 2018). Briefly, master mix solutions (including primers and probes) were treated with 0.5 μl of dsDNase and 0.5 μl of DTT per 20 μl reaction, then incubated at 37°C for 20 min (dsDNase activation), followed by incubation at 60°C for 20 min (dsDNase inactivation). This treatment eliminated amplification of background microbial DNA, preventing negative template controls from reading as positive, and allowing positive/negative calls to be made confidently. Each qPCR was run with three negative template controls and a 1 ng human DNA control (which reflected the average total DNA assay input per sample – combined microbial and human), as human DNA is a known confounder in low biomass 16S rRNA gene studies due to 16S rRNA gene primers/probes binding to human gDNA with varying affinity (Kommedal et al., 2012). For a sample to be called positive, it had to have a cycle threshold (Ct) value at least 1 cycle less than the 1 ng human DNA control that was included on the respective run. Utilizing this conservative approach, we were able to account for mis-priming with human DNA on a run-by-run basis. Samples were screened in duplicate to confirm the findings, and only samples that produced positive results for both replicates were considered positive. All samples were run on a ViiA7 real-time PCR system (Life Technologies).

### *Ureaplasma* Screening

Samples that tested positive for bacterial DNA by 16S rRNA gene qPCR (*n* = 238) were further screened for *Ureaplasma parvum* and *Ureaplasma urealyticum* DNA. Reactions consisted of 6 μl of template or water (negative template control), 1× TaqMan Fast Advanced Master Mix (Applied Biosystems), 0.9 μM of each of the forward (5′-AAGGTCAAGGTATGGAAGATCCAA-3′) and reverse (5′-TTCCTGTTGCCCCTCAGTCT-3′) primers, 0.25 μM of the *U. parvum* and *U. urealyticum* probes (UU-parvo: 5′-FAM-TCCACAAGCTCCAGCAGCAATTTG-BHQ1-3′ and UU-T960: 5′-VIC-ACCACAAGCACCTGCTACGATTTGTTC-BHQ1-3′), and water to a final volume of 20 μL. Positive controls consisting of purified *U. parvum* and *U. urealyticum* DNA were included in each batch.

### Amplification and Barcoding

Amniotic fluid samples from nine women with subsequent spontaneous PTD and 18 matched controls with a delivery at term (all of which were positive for bacterial DNA by qPCR screen) were selected for full-length 16S rRNA gene sequencing. The primers 27F (5′-gcagtcgaacatgtagctgactcaggtcacAGRGTTY GATYMTGGCTCAG-3′) and 1492R (5′-tggatcacttgtgcaagcat cacatcgtagRGYTACCTTGTTACGACTT-3′) were used, with universal UNITAG sequences (in lower case) and a 5′ amine (NH_4_-C_6_) block attached to each primer. A set of three barcoded UNITAG-F and 15 barcoded UNITAG-R primers were designed to generate PacBio sequencing-ready amplicons, using an asymmetric barcoding strategy. All primers were synthesized and HPLC-purified by Integrated DNA Technologies.

To obtain barcoded 16S rRNA gene amplicons, amplification was carried out in two steps. The first PCR was carried out in 50 μL reactions containing 7.5 μL of template or water (negative template control), 1× AccuStart II ToughMix (Quantabio), 0.3 μM each of the forward and reverse primers, 1.25 μL each of the ArcticZymes dsDNase and DTT, and 13.5 μL of water. The PCR amplification program consisted of an initial heating step at 94°C for 3 min; 40 cycles of 94°C for 30 s, 55°C for 30 s, and 72°C for 2 min; and a final extension step of 72°C for 7 min. PCR reactions were performed in a Veriti Thermal Cycler (Applied Biosystems). PCR products were visualized on a QIAXcel automated electrophoresis system using a DNA high resolution gel cartridge (run parameters OM500) to confirm the presence and size of amplicons.

Primary PCR products were purified using NucleoMag NGS magnetic beads (Macherey-Nagel), normalized to 1 ng/μL, then used as template in a secondary, nested PCR, in order to generate asymmetrically barcoded amplicons. Secondary PCR was carried out in 25 μL reactions containing 2 μL of template or water (negative template control), 1× AccuStart II ToughMix, 0.3 μM each of the forward and reverse primers, and 3 μL of water. The PCR amplification program consisted of an initial heating step at 94°C for 3 min; 10 cycles of 94°C for 30 s, 55°C for 30 s, and 72°C for 2 min; and a final extension step of 72°C for 7 min.

### PacBio Sequencing

Barcoded 16S rRNA gene amplicons obtained from the secondary PCR were pooled in equimolar concentrations based on QIAXcel quantitation of the target band. The pool was then concentrated using NucleoMag NGS magnetic beads and eluted in 50 μL of 1× TE. The pool was next visualized in a 1.2% agarose gel using SYBR Safe DNA stain (Invitrogen) and bands of the appropriate size were excised using a sterile disposable scalpel. Excised bands were purified using a QIAquick Gel Extraction Kit as per manufacturer’s instructions. 500 ng of purified DNA was used for library preparation at the Ramaciotti Centre for Genomics, University of NSW, Sydney, NSW, Australia. Here, SMRTbell adapters were ligated onto barcoded PCR products, and the library was sequenced on a PacBio Sequel system on a single SMRT cell. Sequence files were deposited to NCBI Sequence Read Archive under accession number PRJNA602788.

### Sequence Data Analysis

PacBio raw reads were processed using the SMRT Link Analysis software version 6.0 to obtain demultiplexed circular consensus sequence (CCS) reads with a minimum of three full passes and 99.9% sequence accuracy. Sequence data were processed using the software package GHAP v2.1. GHAP is an in-house amplicon processing pipeline developed by Paul Greenfield (CSIRO, Australia) (Greenfield, 2017) built around tools from USearch (Edgar, 2010) and The Ribosomal Database Project (RDP) (Cole et al., 2014), combined with locally written tools for generating OTU tables. BIOM files (McDonald et al., 2012) generated by GHAP were analyzed using MicrobiomeAnalyst – a web-based tool for statistical and visual analysis of microbiome data (Dhariwal et al., 2017). Reads were initially denoised using GHAP with a minimum number of three reads in a minimum of two samples required to retain an OTU. Alpha diversity was assessed as number of observed OTUs and Chao1 (species level analysis). Beta diversity was assessed using PERMANOVA and unweighted unifrac distances. Differential abundance was calculated univariately. All *p-*values were calculated using student’s *t*-test unless stated otherwise.

### Data Analysis

All qPCR data were analyzed using QuantStudio^TM^ Real-Time PCR Software, version 3.1 (Thermo Fisher Scientific) using default settings (including both threshold and baseline set to AUTO) to generate Ct values. Differences between characteristics of subjects with positive or negative 16S rRNA gene results and differences between cases and controls were analyzed with Mann–Whitney U test for continuous variables, due to non-parametric distribution of the variables, and with χ^2^ test or Fishers exact test (if the expected value in any cell were 5 or less) for dichotomous variables. Analyses were performed with IBM SPSS Statistics, version 25).

## Results and Discussion

### Maternal and Neonatal Characteristics

The medical and demographic details of the women being screened for bacterial DNA are presented in Table 1. Cases of missing data reflect women being lost to follow-up, women who had an abortion or miscarriage, and women who were excluded post-enrollment.

**TABLE 1:** Pregnancy demographic characteristics of the cohort (*n* = 1198).

**Characteristic**	**Data available for *n***	**Mean (range) or *n* (%)**
Maternal age (y)	1198	36.4 (20–47)
Nulliparity	1194	319 (27%)
Preterm delivery	1159	78 (7%)
GA at delivery (week^day^)	1159	39^+4^ (22^+1^–43^+0^)
GA at sampling (week^day^)	1193	15^+6^ (13^+6^–22^+1^)
BMI at first prenatal visit	1171	24.5 (15.8–47.4)
Smoking at first prenatal visit	1189	62 (5.2%)
Diabetes type 2	1198	4 (0.33%)
Gestational diabetes	1198	15 (1.3%)
Male/female fetus ratio	1160	592/606 (49.4/50.6%)

*Values are reported as mean (range) or *n* (percent).*

### Bacterial DNA Is Present in Amniotic Fluid in Mid-Pregnancy

Of the 1198 mid-trimester amniotic fluid samples screened by qPCR, 238 (19.9%) contained detectable levels of bacterial DNA. Each of these samples was confirmed as positive for bacterial DNA in duplicate screening. Pregnancy demographic characteristics for 16S rRNA gene positive (*n* = 238) and negative (*n* = 960) samples are presented in Table 2, with no significant differences between the groups. As in Table 1, missing data is due to lost to follow-up, women with an abortion or miscarriage and women who were excluded post-enrollment. Although bacterial load was not quantified in this study, previous reports suggest that amniotic fluid contains 100–1000 16S rDNA copies/mL (Urushiyama et al., 2017; Lim et al., 2018). Considering that there is substantial 16S rRNA gene copy number variation within the genomes of different bacterial species however, these estimates do not correlate with CFU/mL and actual bacterial cell numbers may be as low as 1/15th of these values, depending on the bacterial species present (Kembel et al., 2012). Regardless, these data emphasize the low biomass nature of this sample type. In the present study, the average Ct value for the 16S rRNA gene positive samples was 34.1 (range: 28.5–43.9). Using a previously described enzymatic decontamination protocol (Stinson et al., 2018), we were able to produce completely negative extraction and PCR controls in our qPCR screen, and this, combined with the use of a biologically relevant (in terms of quantity) human DNA control with each run to account for any potential mis-priming, allowed us to make positive/negative calls with a higher level of sensitivity and accuracy. It should be noted that we did not collect negative controls during sampling, and so we are not able to assess the possibility of contamination during sample collection and initial processing, a limitation that is common in most microbiome studies. However, the absence of detectable bacterial DNA in the majority of samples suggests that our sample collection protocol did not introduce ubiquitous contamination.

**TABLE 2:** Pregnancy demographic characteristics for 16S rRNA gene positive (*n* = 238) and negative (*n* = 960) samples.

**Characteristic**	***n***	**Positive**	**Negative**	***p*-Value**
Maternal age (y)	1198	37 (21–46)	37 (20–47)	0.90
Nulliparity	1194	65 (27%)	254 (27%)	0.81
Preterm delivery	1159	64 (7%)	14 (6%)	0.68
GA at delivery (week^+day^)	1159	39^+4^ (30^+5^–43^+0^)	39^+3^ (22^+1^–43^+0^)	0.36
GA at sampling (week^+day^)	1193	15^+6^ (14^+2^–20^+4^)	15^+6^ (13^+6^–22^+1^)	0.47
BMI at first prenatal visit	1171	23.1 (16.9–45.4)	23.6 (15.8–47.4)	0.18
Smoking at first prenatal visit	1189	15 (6%)	47 (5%)	0.38
Diabetes type 2	1198	4 (0.4%)	0 (0)%	1.0
Gestational diabetes	1198	1 (0.%)	14 (1.5%)	0.33
Male/female fetus ratio	1160	111/118 (48.5/51.5%)	481/450 (51.7/48.3%)	0.39

*Values are reported as mean (range) or *n* (percent).*

We are not the first to screen amniotic fluid samples from mid-trimester using universal 16S rRNA gene primers. Two previous such studies have reported that 0 and 3% of samples were positive for bacterial DNA (Rowlands et al., 2017; Kayem et al., 2018). Neither of these studies, however, reported any details of controls used and in the case of Rowlands et al. (2017), who used a qPCR assay, it is unclear if samples were truly negative or if Ct cut-off value for a negative result was set based upon amplification present in negative PCR controls (a common practice used for distinguishing “positive” samples from background noise). Without the use of decontaminated reagents, negative controls frequently appear at Cts similar to those of amniotic fluid in 16S rRNA gene qPCR screening tests, making it incredibly difficult to determine a true positive result.

Interestingly, our detection of bacterial DNA in the amniotic fluid was not associated with spontaneous PTD. Only 14 of the 238 positive samples (6.1%) were from women with a spontaneous PTD, four of which were iatrogenic (medically indicated) PTD. Of the samples that tested negative for bacterial DNA, 6.9% were from women with a spontaneous PTD. There was also no difference in the average gestational age at sampling for those that tested positive for bacterial DNA compared to those that tested negative (*p* = 0.47). Our data indicate that in some pregnancies, bacterial DNA is present in the amniotic fluid at 14–20 weeks gestation. However, in the majority of cases, the amniotic fluid appears sterile at this gestational age. Presence of bacterial DNA in the amniotic cavity at mid-trimester did not predict spontaneous PTD in our cohort.

Demographic details of the cases and controls which were selected for bacterial profiling are presented in Table 3. There were no significant differences between the groups.

**TABLE 3:** Pregnancy demographic characteristics for cases (spontaneous preterm delivery (<37 gestational weeks; *n* = 9) and controls (delivery at term; *n* = 18).

**Characteristic**	**Cases (*n* = 9)**	**Controls (*n* = 18)**	***p*-Value**
Maternal age (y)	36 (21–43)	36 (23–44)	0.94
Nulliparity^a^	2 (22%)	4 (22%)	1.00
GA at delivery (week^+day^)	34^+6^ (30^+5^–36^+6^)	39^+3^ (37^+1^–42^+1^)	<0.001
GA at sampling (week^+day^)	15^+4^ (14^+4^–16^+5^)	15^+5^ (14^+2^–16^+5^)	0.78
Pre-pregnancy BMI^a^	24 (21–27)	24 (19–29)	0.78
Smoking during pregnancy^a^	0 (0%)	0 (0%)	–
IVF^a^	1 (11%)	2 (22%)	1.00
Diabetes type 2	0 (0%)	0 (0%)	–
Gestational diabetes	0 (0%)	0 (0%)	–
Male/female fetus ratio	4/5 (44/56%)	7/11 (39/61%)	1.00

*Values are reported as mean (range) or *n* (percent). ^*a*^Matching criteria.*

The potential exposure of the fetus to bacteria at this early gestational age is of particular interest, as the fetal skin does not keratinize until approximately 20 weeks gestation (Wiberg-Itzel, 2012). Prior to this stage, the fetal skin offers no resistance to movement of the amniotic fluid, which can be thought of as an extension of the fetal extra-cellular fluid. After fetal skin keratinization occurs, the amniotic fluid changes in osmolality and can no longer equilibrate with the fetus. The effect that fetal skin keratinization has on the amniotic fluid microbiome is unknown. The samples examined in the present study were primarily taken prior to gestational week 20. The fetal skin would not have acted as an effective barrier between the amniotic fluid microbiome and the fetal extracellular fluid at this stage in development.

### *Ureaplasma* spp. Were Not Detected in Mid-Trimester Amniotic Fluid Samples

*Ureaplasma parvum* and *Ureaplasma urealyticum* infection of the intra-amniotic cavity is typically associated with spontaneous PTD (Gray et al., 1992; Cassell et al., 1993; Horowitz et al., 1995). Therefore, all samples that tested positive for bacterial DNA by 16S rRNA gene qPCR (using primers optimized to improve coverage of *Ureaplasma* spp.) were further screened for *U. parvum* and *U. urealyticum* DNA using targeted qPCR (Yi et al., 2005). All of these were found to be negative. Further, no members of the *Ureaplasma* genus were identified by 16S rRNA gene sequencing. These results are similar to those reported by Payne et al. (2014) and Rowlands et al. (2017), both of whom failed to detect *Ureaplasma* spp. DNA in mid-trimester amniotic fluid from Australian women. Payne et al. (2014), however, did detect *Ureaplasma* spp. DNA in two women (0.42%) from their Chinese cohort. In contrast, Kayem et al. (2018) screened 980 amniotic fluid samples taken for genetic testing at 16–20 weeks gestation and reported that 12 samples (1.22%) were positive for *Ureaplasma* spp. DNA (10 *U. urealyticum* and 2 *U. parvum*) by qPCR screening. Collectively the evidence suggests that *Ureaplasma* spp. colonization of the amniotic cavity in mid-gestation is a rare event.

### Description of Bacterial DNA Profiles in Mid-Trimester Amniotic Fluid

**FIGURE 2:**
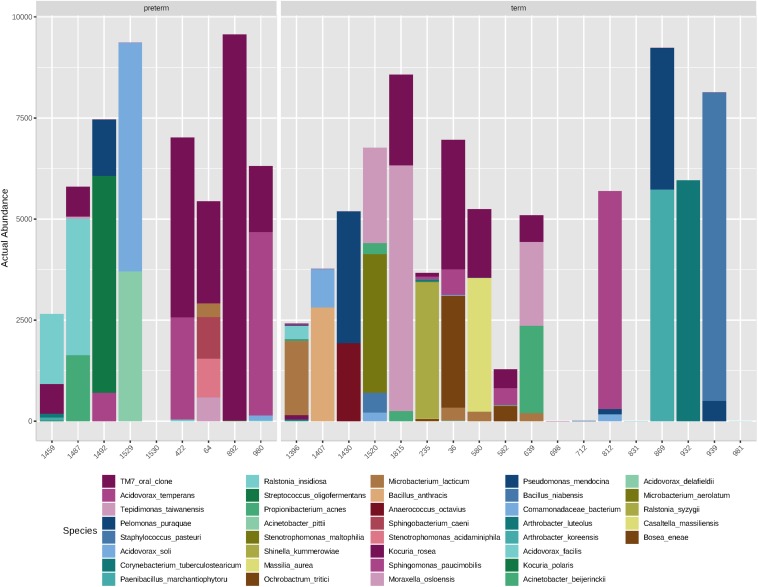
Abundance (number of reads) of bacterial species detected in mid-trimester amniotic fluid samples from women with spontaneous preterm delivery (cases, *n* = 9) and women with a delivery at term (matched controls, *n* = 18).

Overall, the amniotic fluid samples profiled here contained low-abundance and low-diversity bacterial DNA profiles, as was expected based on previous studies (Collado et al., 2016; Urushiyama et al., 2017; Lim et al., 2018; Wang et al., 2018; Figure 2). Of the 27 amniotic fluid samples sequenced, five (one case and four controls) returned insufficient reads (≤50). Each of these samples gave a clear positive signal on the qPCR screen, but failed to produce an amplicon with the full-length 16S rRNA gene primers used for sequencing, suggesting that the bacterial DNA present in these samples were not covered by these primers. This raises the possibility that the full breadth of the amniotic fluid bacterial communities present were not captured in this study. Of the remaining samples, we recovered an average of 6503 reads per sample (range 1284–9563) and 12 OTUs per sample (range 2–33). Here, our negative PCR controls were completely negative, while our negative ECs were sporadically positive. Seven taxa were detected in six ECs: *Pelomonas puraquae*, *Casaltella massiliensis*, *Staphylococcus pasteuri*, *Bosea eneae*, *Bosea vestrisii*, *Acinetobacter beijerinckii*, and *Rhodobacter blasticus* (Table 4). However, in cases where one of these OTUs was detected in an EC, it was not detected in the samples that were extracted in that batch, suggesting that extraction-based contamination was not a contributor to our results.

**TABLE 4:** Bacterial taxa (number of reads) detected in negative extraction controls (EC) and negative PCR controls (NTC, no template controls).

**Species**	**EC1**	**EC2**	**EC5**	**EC6**	**EC7**	**EC8**	**EC10**	**EC11**	**NTC1**	**NTC2**
*Casaltella massiliensis*					10170					
*Pelomonas puraquae*	6075									
*Bosea eneae*							1719			
*Bosea vestrisii*							1338			
*Acinetobacter beijerinckii*		2236								
*Staphylococcus pasteuri*						208		601		
*Rhodobacter blasticus*						525				

**FIGURE 3:**
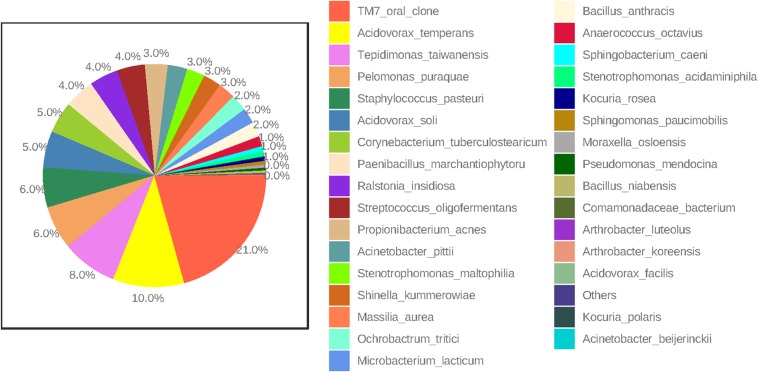
Percent abundance (highest to lowest) of bacterial species in all mid-trimester amniotic fluid samples (*n* = 27).

At the species level, amniotic fluid samples were dominated by reads that mapped to Saccharibacteria (TM7 oral clone), *Acidovorax temperans*, *Tepidimonas taiwanensis*, and *P. puraquae* (Figure 3). Eleven samples contained reads that mapped to Saccharibacteria (TM7 human oral clone), an extremely small coccus (200–300 nm) that until recently has been uncultivated, but is ubiquitous in numerous environments (He et al., 2015). It is important to note that Saccharibacteria was not a particularly good match for this particular OTU, with only 92.6–93% sequence homology. This might indicate that these OTUs belong not to Saccharibacteria, but to a related, as-yet undescribed bacteria (for example, an uncultivated clade of TM7). Six samples contained reads that mapped to *A. temperans* with a reasonably good level of sequence homology (98.3%). *A. temperans* is a Gram-negative rod with mega-pili on its surface, and has previously been isolated from various clinical and environmental samples, including urine, cervical swabs, and wastewater (Willems et al., 1990). This species has not previously been identified in amniotic fluid. Four samples contained reads that mapped to *T. taiwanensis* with 97.2% sequence homology. *T. taiwanensis* is a relatively uncharacterized bacteria that was first described in 2006 in hot springs (Chen et al., 2006). It is a Gram-negative, motile rod with a single polar flagellum. Given that *T. taiwanensis* is a thermophile found at moderately high temperatures (50–60°C), it is unlikely to be the correct identity for this particular OTU. Although the sequence matched reasonably well to this particular bacterium, a 2.8% difference in a 1.5 kb amplicon represents a 42 bp difference in the 16S rRNA gene. It is, therefore, possible that this OTU belongs to an as yet uncharacterized bacterium. This sequence was not detected in any of the negative controls. Four samples contained reads that mapped to *P. puraquae* with a high level of sequence homology (99.5%). *P. puraquae* is a relatively uncharacterized bacteria, first isolated in 2007 from hemodialysis water (Gomila et al., 2007), and previously reported in amniotic fluid samples collected during cesarean section (Stinson et al., 2019a) and in endometrial samples (Fang et al., 2016; Verstraelen et al., 2016). It is a Gram-negative, motile rod with a single polar flagellum. Although *P. puraquae* is often thought of as a contaminant in microbiome research (Salter et al., 2014), it was present in only one of the eight ECs taken here and in neither of the two PCR controls. The ECs processed in the same batch as the samples that contained reads mapping to *P. puraquae* did not contain reads mapping to *P. puraquae* themselves. Although this species has not previously been identified in mid-trimester amniotic fluid samples, Urushiyama et al. (2017) reported finding reads that mapped to *Pelomonas saccharophila* in mid-trimester amniotic fluid samples using short read sequencing.

Other less abundant OTUs identified here are in line with those previously documented in mid-trimester amniotic fluid samples by others. Four of our samples contained reads that mapped to *Propionibacterium acnes*, which has previously been identified in mid-trimester amniotic fluid (Urushiyama et al., 2017; Zhu et al., 2018) as well as in amniotic fluid at term (Collado et al., 2016; Stinson et al., 2019a). We also identified reads that mapped to *Bacillus* sp. (which could not be differentiated between *Bacillus anthracis*, *Bacillus cereus*, *Bacillus mycoides*, *Bacillus pseudomycoides*, *Bacillus thuringiensis*, and *Bacillus weihenstephanensis*) and *Bacillus niabensis*. Reads that map to species of the *Bacillus* genus have been consistently reported in previous studies of the mid-trimester amniotic fluid microbiome (Urushiyama et al., 2017; Kayem et al., 2018; Zhu et al., 2018). Other genera detected here that have also been detected using short sequence technology in mid-trimester amniotic fluid include *Arthrobacter* sp., *Streptococcus* sp., *Staphylococcus* sp., *Massilia* sp., and *Paenibacillus* sp. (Urushiyama et al., 2017; Kayem et al., 2018; Zhu et al., 2018). Interestingly, bacterial species typically associated with ascending intrauterine infection and preterm labor were not detected in this study (Keelan et al., 2016). This supports the view that invasion by such species represents a pathological event that likely occurs after 20 weeks’ gestation, whereas potential colonization by the species identified in this study occurs much earlier and is non-pathogenic.

### Comparison of Mid-Trimester Bacterial DNA Profiles From Term and Preterm Pregnancies

**FIGURE 4:**
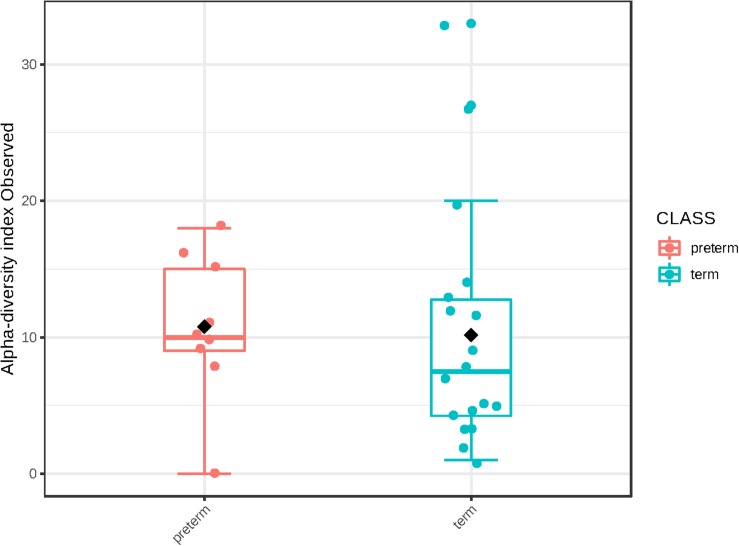
Alpha diversity (number of observed operational taxonomic units) in cases (*n* = 9) and controls (*n* = 18).

We were unable to identify any significant differences in bacterial DNA profiles of amniotic fluid from women with a spontaneous PTD or a delivery at term. There was no difference in alpha diversity of cases and controls (number of observed OTUs *p* = 0.87; Chao1 *p* = 0.44) (Figure 4). Nor was there any difference in beta diversity of cases and controls (PERMANOVA *p* = 0.534). None of the species detected here varied in abundance between cases and controls upon univariate analysis.

We may have been unable to identify any predictive bacterial DNA signatures for spontaneous PTD due to the gestational age at which these samples were taken. Spontaneous PTD-associated bacteria have previously been detected in amniotic fluid samples early in gestation [*Ureaplasma* spp. at 20 weeks (Han et al., 2009; Romero et al., 2014), *Fusobacterium* spp. at 21 weeks (Han et al., 2009), and *U. parvum*, *Citrobacter koseri* and viridans group streptococcus at 22 weeks (Han et al., 2009; Romero et al., 2014)], but not prior to 20 weeks. The average gestational age at which the present samples were taken was 15^+6^ weeks. It may have been more informative for our purposes to collect amniotic fluid samples later in the second trimester; however, amniocentesis is not routinely performed at this stage of pregnancy, making such a study unfeasible. Further, ours was a cohort of asymptomatic women presenting for genetic amniocentesis, while previous detection of early pathogenic colonization of amniotic fluid has occurred in women presenting with spontaneous preterm labor and preterm rupture of membranes.

Previously, Urushiyama et al. (2017) profiled amniotic fluid samples obtained during cesarean section from pregnancies with various stages of chorioamnionitis. As well as identifying pathogenic bacteria in cases of high grade chorioamnionitis, these authors also found an inverse relationship between alpha diversity and chorioamnionitis severity. This might suggest that amniotic fluid with a low diversity of bacteria is more vulnerable to infection than amniotic fluid with a high diversity of bacteria. Conversely, these data might also suggest that intra-amniotic infection with pathogenic bacteria reduces amniotic fluid diversity. Their data therefore poses a chicken or the egg question: Does low diversity precede infection, or does infection result in low diversity? We did not find any differences in the alpha diversity of amniotic fluid samples from women with a spontaneous PTD compared to women with a term delivery. However, given the low number of spontaneous PTD in our cohort and the lack of data on placental pathology, we are unable to clarify this question here.

### Contribution to the “Sterile Womb” Debate

The idea that the womb is sterile continues to be hotly debated in both scientific and lay circles. The data presented here at the very least support the notion that exposure to bacterial DNA may occur prior to birth in some healthy pregnancies. Further, we have provided evidence that this exposure occurs early in pregnancy. Although we do not know if this DNA is representative of live, biologically active cells, due to our carefully controlled processing protocols, it is unlikely to be cell-free bacterial DNA. However, questions remain around the interpretation of such data. Low biomass samples are notoriously difficult to accurately characterize, and the interpretation of data from these environments must be performed cautiously (Stinson et al., 2019b). As in all other microbiome studies, there is also always a risk of contamination. Although we have used rigorous experimental controls and decontaminated PCR reagents, it should be kept in mind that we did not include any negative controls during sampling. Further, the observed bacterial signatures in these samples do not necessarily imply the presence of a true “microbiome” (that is, an ecological system of interacting organisms and their environment). Our results might reflect the presence of bacteria that are transferred from mother to fetus, but are not suited to survive within the womb. These bacteria may therefore be transiently present, but not true colonizers. Indeed, this may explain the low abundance of bacteria in our samples. If a true microbiome was established this early in pregnancy, we might expect it to become increasingly abundant after months of incubation *in utero*. However, data from other studies of the amniotic fluid microbiome suggest that it remains low abundance at term (Collado et al., 2016; Urushiyama et al., 2017; Stinson et al., 2019a). Another explanation for the low titer of bacteria found within the intra-amniotic space is the presence of both fetal and maternal immunological components in amniotic fluid which may suppress bacterial growth [for instance maternal and fetal neutrophils (Gomez-Lopez et al., 2017)]. Alternatively, non-bacterial microbial elements such as bacteriophages may curate bacterial communities *in utero* and prevent them from expanding in biomass; a previous study has already reported that nearly all reads associated with viruses in amniotic fluid were associated with bacteriophages (Lim et al., 2018).

## Conclusion

Here we have provided evidence that, while most mid-trimester amniotic fluid samples are sterile, bacterial DNA is present in amniotic fluid at this time point in approximately one in five pregnancies. Mid-trimester amniotic fluid bacterial DNA profiles are of low biomass and low diversity. We were not able to identify any differences in the bacterial DNA profiles of samples from pregnancies that had a spontaneous PTD and matched term controls. The developmental and immunological significance of the presence or absence of bacterial DNA in mid-trimester amniotic fluid samples remains unclear.

## Data Availability Statement

The datasets generated for this study can be found in the PRJNA602788.

## Ethics Statement

The study was approved by the Ethics Review Board at the University of Gothenburg, Sweden (Dnr Ö 639-03, T 318-08, T 694-11 and Dnr 2019-0602) and written informed consent was obtained from all participants.

## Author Contributions

BJ and MH designed the study and were responsible for sample collection. MH and FV collected the data. MB and LS performed the statistical analyzes and graphical presentation of data. LS performed the DNA extractions, PCR, sequencing, microbiome data analysis, and drafted the manuscript. BJ, MH, and MP received funding for the project. MP, JK, MK, and BJ supervised the work. All authors participated in the planning stages of analyzes and interpretation of data, and contributed to the manuscript by critical review and editions.

## Conflict of Interest

The authors declare that the research was conducted in the absence of any commercial or financial relationships that could be construed as a potential conflict of interest.
